# Physics-Informed Neural Networks for Modeling Physiological Time Series: A Case Study with Continuous Blood Pressure

**DOI:** 10.21203/rs.3.rs-2423200/v1

**Published:** 2023-01-16

**Authors:** Kaan Sel, Amirmohammad Mohammadi, Roderic I. Pettigrew, Roozbeh Jafari

**Affiliations:** 1 Department of Electrical and Computer Engineering, Texas A&M University, College Station, TX, USA; 2 Department of Computer Science and Engineering, Texas A&M University, College Station, TX, USA; 3 School of Engineering Medicine, Texas A&M University, Houston, TX, USA; 4 Department of Biomedical Engineering, Texas A&M University, College Station, TX, USA

## Abstract

The bold vision of AI-driven pervasive physiological monitoring, through the proliferation of off-the-shelf wearables that began a decade ago, has created immense opportunities to extract actionable information for precision medicine. These AI algorithms model the input-output relationships of a system that, in many cases, exhibits complex nature and personalization requirements. A particular example is cuffless blood pressure estimation using wearable bioimpedance. However, these algorithms need to be trained with a significant amount of ground truth data. In the context of biomedical applications, collecting ground truth data, particularly at the personalized level is challenging, burdensome, and in some cases infeasible. Our objective is to establish physics-informed neural network (PINN) models for physiological time series data that would reduce reliance on ground truth information. We achieve this by building Taylor’s approximation for the gradually changing known cardiovascular relationships between input and output (e.g., sensor measurements to blood pressure) and incorporating this approximation into our proposed neural network training. The effectiveness of the framework is demonstrated through a case study: continuous cuffless BP estimation from time series bioimpedance data. We show that by using PINNs over the state-of-the-art time series regression models tested on the same datasets, we retain a high correlation (systolic: 0.90, diastolic: 0.89) and low error (systolic: 1.3 ± 7.6 mmHg, diastolic: 0.6 ± 6.4 mmHg) while reducing the amount of ground truth training data on average by a factor of 15. This could be helpful in developing future AI algorithms to help interpret pervasive physiologic data using minimal amount of training data.

AI algorithms provide unique opportunities for extracting complex actionable information from wearable physiological time series data for precision medicine^1^. These techniques, in particular machine learning (ML) and deep learning (DL) approaches, model the input-output relationships of the system, where in many cases, this system exhibits a complex nature and personalized requirements, e.g., cuffless blood pressure (BP) estimation using wearable bioimpedance^2,3^. ML- and DL-based modeling requires significant amounts of personalized ground truth data for training^4^. Collecting this ground truth data for each individual often requires the use of invasive or obtrusive medical-grade measurement systems (e.g., arterial line or cuff-based sphygmomanometer for peripheral BP monitoring), and therefore is challenging, burdensome, and in some cases infeasible. To address this gap, we need to establish high-fidelity learning models for time series data that rely on reduced amounts of ground truth data.

For many engineering or biological systems, there exists a vast body of domain knowledge that may be leveraged in training deep neural networks (DNNs) to reduce the reliance on ground truth (i.e., labeled) data. A promising direction is the construction of physics-informed neural networks (PINNs) ^5^, where the neural networks are trained to solve scientific problems leveraging underlying physics laws structured by generalizable nonlinear partial differential equations (PDEs). This is achieved by augmenting neural network training with a unique loss function that includes these PDEs in addition to the standard supervised loss. Therefore, during training, PINN weights are optimized to minimize the loss function that accounts for additional physical constraints. PINNs have proven to be highly effective in solving many complex engineering problems (e.g., fluid mechanics^6^, cyber-physical systems^7^, power systems^8^, molecular biology^9^) with limited experimental data. However, given the inter-subject variations in the cardiovascular system, the relationships that connect wearable measurements to cardiovascular parameters are not well-defined in the form of generalized PDEs^10,11^. For example, using hand-crafted equations defined between cardiovascular parameters and wearable time series data is infeasible since; (i) the features of these wearable measurements provide a proxy for physiological parameters that are not generalizable, e.g., pulse transit time-based BP estimation requiring frequent calibration due to its dependency on personalized arterial properties that are not accessible with wearables^12^, (ii) these equations fail to track and adapt to time-dependent changes in cardiovascular dynamics e.g., increasing BP, with heart rate increasing due to increased vagal tone or decreasing due to increased sympathetic activity^13–15^. Therefore, there is an unmet need to establish new ways to leverage PINNs for time series data in cardiovascular applications.

In this work, we propose a novel approach to establish PINNs for extracting essential cardiovascular parameters (e.g., BP) from physiological time series data with limited use of ground truth data. We achieve this by building Taylor’s approximation for certain gradually changing cardiovascular phenomena, e.g., establishing the relationship between physiological features extracted from bioimpedance sensor measurements and BP. This approximation yields a Taylor approximation polynomial in the form of a PDE that includes partial derivatives (i.e., gradients) of the output with respect to the input. The values of these gradients are obtained with auto-differentiation that is inherently available in neural networks. We calculate a remainder term (i.e., residual or physics-based loss) from the difference between Taylor’s approximation and the neural network predictions and include it in the model loss function. This enables the optimization of neural network weights based on the total loss function resulting from the sum of the standard loss and the residual. The Taylor polynomial can be used to generate an approximation of the output for any input, without the use of the corresponding true labels. Therefore, the physics-based loss can be calculated for neural network predictions for all inputs. This would lead to obtaining predictions that show minimal deviation from the approximated Taylor polynomial.

The effectiveness of the framework is demonstrated through a comprehensive case study on continuous cuffless BP estimation from wearable time series bioimpedance data. BP is a significant cardiovascular parameter frequently used by clinicians to assess cardiac and circulatory health along with their associated risk factors and disorders^16–19^. Conventional BP measurements – yielding systolic (SBP), diastolic (DBP), and pulse pressure (PP) values – are based on oscillometric cuff inflation/deflation which causes discomfort, precluding frequent use^20^. Therefore, this case study; (i) includes a cuffless wearable BP estimation scenario that generates time series data, e.g., bioimpedance, (ii) targets a problem with clinical importance, (iii) requires the acquisition of ground truth data that is challenging to obtain, (iv) utilizes complex cardiovascular dynamics driving the translation of wearable bioimpedance into BP^21–23^. We focus on a single wearable modality (e.g. bioimpedance) for our time series measurements to ensure that the underlying physics remain consistent for one modality (see Methods and Supplementary Text 1 for additional details on bioimpedance).

Figure 1a shows a high-level representation of the proposed framework demonstrated through the selected case study. The input features for our models are extracted from non-invasive bioimpedance sensors placed at the participants’ wrists or fingers in a wearable form factor (see Supplementary Figure 1). We evaluated the accuracy of the PINN model predictions on different datasets having N=15 participants, who went through various BP elevation maneuvers (e.g., hand gripper exercise, cold-pressor test), achieving a wide range of pressure values (0.04–0.96 quantiles, systolic: 104–205 mmHg, diastolic: 51–136 mmHg, pulse pressure: 29–103 mmHg). The proposed PINNs retain high accuracy (ME ± SDE, systolic: 1.3 ± 7.6 mmHg, diastolic: 0.6 ± 6.4 mmHg, pulse pressure: 2.2 ± 6.1 mmHg), while decreasing the required amount of ground truth data, on average, by a factor of 15, based on the comparison with the state-of-the-art time series regression models (see Supplementary Tables 1–3). We provided an additional proof-of-concept study to show that the optimized PINN models demonstrate a consistent approximation of the input-output relationship for varying amounts of training ground truth data.

## Results

### Physics-informed neural network model for cuffless BP estimation

#### PINN model design.

A conventional deep neural network (DNN) is trained through supervised learning – also known as inductive learning - where model weights are optimized based on a loss function that uses a labeled training dataset (i.e., the true output is known). PINN models are transductive, meaning that in contrast to inductive learning, the models make use of additional information present in the unlabeled (i.e., the true output is unknown) input data^24^. This information is integrated to the model through a modification of the conventional loss function, ${ℒ}_{conventional}$, which is originally calculated using the model predictions and true output labels. In contrast, the modified loss function includes an additional loss term, ${ℒ}_{physics}$, as shown in Eq. (1).

(1) $${ℒ}_{total}={ℒ}_{conventional}+{ℒ}_{physics}$$

**Figure 1.**
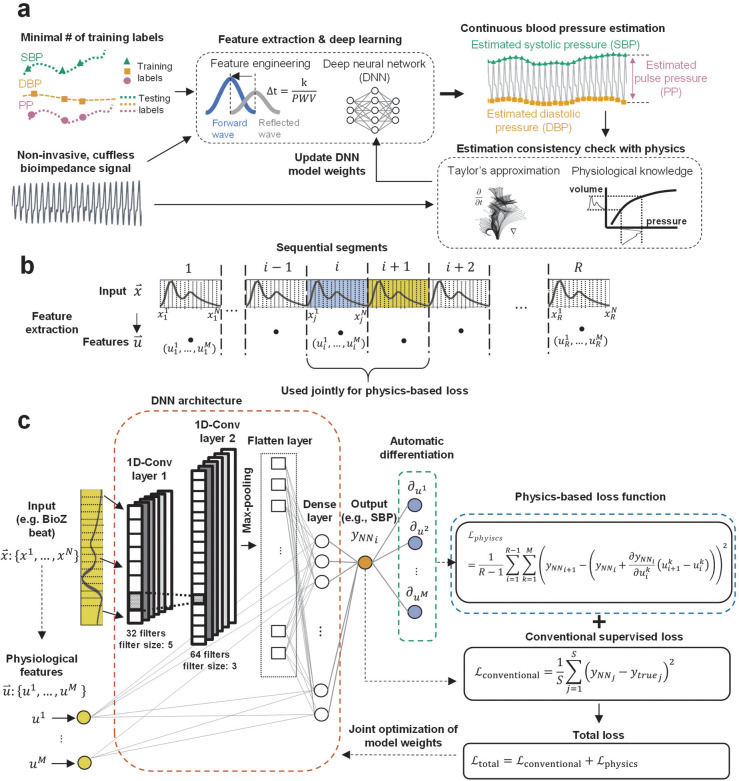
Physics-informed neural network (PINN). **a.** The deep neural network (DNN) model uses input time series measurements (e.g. bioimpedance, BioZ) to estimate continuous systolic, diastolic, and pulse pressure values. Taylor’s approximation is defined for physiological features extracted from BioZ, and BP is used to guide neural network training. The parameters of the approximation are calculated with DNN auto-differentiation of predictions with respect to input features. This approximation is compared with the DNN predictions to estimate values for the physics-based loss function. **b.** The definition of indexes for the sequential segmented input bioimpedance data, $\vec{x}$, having N sample points per segment, and the extracted feature set, $\vec{u}$ with M dimensions (i.e., number of features). **c.** DNN architecture for PINN models. We use convolutional neural network (CNN) layers extracting information from segmented BioZ, concatenate the output with the physiological features extracted from each segment, and estimate BP, *y*_*NN*_. A conventional loss function, *e*_conventional_, is calculated over a labeled set of size *S*, based on the model output and true BP labels (*y*_*true*_). This value is used for conventional neural network optimization. For PINNs, we additionally calculate the partial differentials of *y*_*NN*_ with regards to the physiological input features and fit it into Taylor’s approximation polynomial. This polynomial is constructed for each input segment from the dataset of size *R* and evaluated at the next consecutive segment. We calculate mean squared error from the difference between Taylor approximations and the corresponding neural network predictions. This new error function combined with the conventional error function are used to train the PINN models.

Here, ${ℒ}_{total}$ represents the modified model loss function due to the addition of ${ℒ}_{physics}$ to conventional supervised loss. Note that an inaccurate definition of ${ℒ}_{physics}$ may degrade model accuracy as it may lead to misguided weight optimization. We construct ${ℒ}_{physics}$ based on Taylor’s approximation of the known physiological dynamics driving the translation of bioimpedance to BP, e.g., hemodynamic relationships defined between blood volume, arterial compliance, and BP^21,25^: let ${\vec{x}}_{i}:\left\{x_{i}^{1},\ldots ,x_{i}^{N}\right\}$ be the *N* dimensional time series bioimpedance data segmented based on the start and end of the *i*-th cardiac cycle and sampled to have *N* points for each segment, ${\vec{u}}_{i}:\left\{u_{i}^{1},\ldots ,u_{i}^{M}\right\}$ be the *M* dimensional vector corresponding to *M* features calculated from ${\vec{x}}_{i}$, and Θ denote the neural network weights (Figure 1b). The neural network generates an output, *y*_*NN*_ based on $\vec{x}$, $\vec{u}$, and Θ, such that $y_{NN}=f_{NN}(\vec{x},\vec{u},\Theta )$. Here, *f*_*NN*_ is the approximated function by the neural network. Using the feature vector $\vec{u}$, we define a polynomial with Taylor’s approximation around *i*-th segment as shown in Eq. (2).

(2) $$P_{i}(\vec{x},\vec{u},\Theta )=f_{NN}\left({\vec{x}}_{i},{\vec{u}}_{i},\Theta \right)+{\sum_{k=1}^{M}\frac{\partial f_{NN}}{\partial u^{k}}|}_{i–\text{th}\;\text{segment}}\left(u^{k}-u_{i}^{k}\right)$$

$P_{i}(\vec{x},\vec{u},\Theta )$ represents this Taylor polynomial approximated based on *i*-th segment. *∂f*_*NN*_/*∂u*^*k*^|_*i*–th segment_ is calculated with neural network auto-differentiation for the *i*-th segment. The output of this polynomial can be evaluated for any given $\vec{x}$, $\vec{u}$ pair and neural network weights. Note that the bioimpedance and blood pressure data are sequential, e.g., *i*-th and (*i*+1)-th segments represent consecutive beats. We leverage this sequential nature of our data by evaluating Taylor polynomials approximated around *i*-th segment for input values at (*i*+1)-th segment, i.e., $P_{i}\left({\vec{x}}_{i+1},{\vec{u}}_{i+1},\Theta \right)$. Next, we calculate a residual resulting from the difference between the neural network prediction and the Taylor polynomial evaluated at the (*i*+1)-th segment as shown in Eq. (3).

(3) $$f_{NN}\left({\vec{x}}_{i+1},{\vec{u}}_{i+1},\Theta \right)=P_{i}\left({\vec{x}}_{i+1},{\vec{u}}_{i+1},\Theta \right)\;\;+h_{i}\left({\vec{x}}_{i+1},{\vec{u}}_{i+1},\Theta \right),$$

Here, $h_{i}\left({\vec{x}}_{i+1},{\vec{u}}_{i+1},\Theta \right)$ denotes this residual value evaluated at (*i*+1)-th segment using Taylor’s approximation around *i*-th segment, such that,

(4) $$\underset{\begin{array}{c} {\vec{x}}_{i+1}\to {\vec{x}}_{i,}\\ {\vec{u}}_{i+1}\to {\vec{u}}_{i} \end{array}}{\lim}h_{i}\left({\vec{u}}_{i+1},{\vec{u}}_{i+1},\Theta \right)=0$$

The value of *h* represents a physics-based loss for the neural network. Given that *h* is calculated unsupervised (i.e., labels of output are not used), we can calculate *h* for any given input sequence. We evaluate the value of *h* for all consecutive input segments and use the mean squared sum of this evaluation for the physics-based loss function, as shown in Eq. (5).

(5) $${ℒ}_{physics}=\frac{1}{\left(R-1\right)}\sum_{i=1}^{R-1}{\left(h_{i}\left({\vec{x}}_{i+1},{\vec{u}}_{i+1},\Theta \right)\right)}^{2}$$

where *R* is the total number of segments. The conventional loss function, however, only uses the labeled training points, as shown in Eq. (6).

(6) $${ℒ}_{conventional}=\frac{1}{S}\sum_{i=1}^{S}{\left(y_{NN}\left({\vec{z}}_{i},{\vec{x}}_{i};\Theta \right)-y_{true}\left({\vec{z}}_{i},{\vec{x}}_{i}\right)\right)}^{2}$$

where *S* is the number of labeled data instances. The partial derivatives defined within ${ℒ}_{physics}$, i.e., $\partial f_{NN}/\partial \vec{u}$, represents the approximated non-stationary relationship between physiological input features and output BP. The evaluation of ${ℒ}_{physics}$ across the complete unsupervised input set enables neural network predictions to be aware of the input-output relationships approximated with Taylor polynomial. The use of limited supervised points allows the neural network to obtain blood pressure predictions that satisfy the physical constraint defined with ${ℒ}_{physics}$ rather than arbitrary values.

#### PINN model architecture.

To assess the effectiveness of the physics-integration in DNN training, we create an identical DNN architecture and training/testing data for both the proposed PINN and the conventionally trained DNN. The only difference is the definition of the loss functions for these models, i.e., the PINN includes the additional loss term, ${ℒ}_{physics}$. The DNN uses a series of one-dimensional convolutional neural networks (CNN) to extract hidden features and parameters from a given bioimpedance waveform, ${\vec{x}}_{i}$. We concatenate the flattened CNN outputs with neurons connecting physiologically relevant input features, ${\vec{u}}_{i}$, to the DNN architecture. We then add fully connected network (FCN) layers, with the final layer having a single neuron, outputting a prediction of the reference ground truth information, such as BP. Figure 1c shows the proposed model architecture. The details of the model hyperparameters are shared in the Methods section.

#### Definitions of physiologically relevant input features and model output.

The bioimpedance sensors placed along the peripheral (e.g., radial, digital) arteries capture quasi-periodic waveforms. We defined three features (i.e., $\vec{u}:\left\{u^{1},u^{2},u^{3}\right\}$) extracted at every heartbeat window from pre-processed bioimpedance signals based on their physiological relevance to hemodynamic features that exhibit certain relationships with BP. With every heartbeat, a pressure pulse propagates through the arteries causing an expansion in artery volume and an increase in pressure. The change in volume and pressure is based on the elastic wall properties of the arteries. An increase in volume with pulse arrival causes a drop in the bioimpedance amplitude due to the blood’s higher conductivity than surrounding tissue^22^. The first feature, *u*^1^, corresponds to the level of amplitude change in the bioimpedance waveform, providing a proxy to an increase in blood volume^22^. Under varying BP, the arterial wall characteristics also affect the blood pulse wave velocity (PWV). Higher PWV results in the earlier arrival of the reflected pulse wave (e.g., due to arterial tree branching of the radial artery to the digital arteries)^26,27^. Hence, it causes an earlier secondary drop in bioimpedance amplitude when the reflected pulse reaches the arterial site. The second feature, *u*^2^, measures the inverse of the time difference between these two impedance drops, e.g., caused by the arrivals of the systolic and reflection waves, and gives an indirect proxy to PWV and artery elasticity^28,29^. Lastly, the third feature, *u*^3^, is beat-to-beat heart rate (HR), measured from the time difference between the end and the beginning of the waveform.

**Figure 2.**
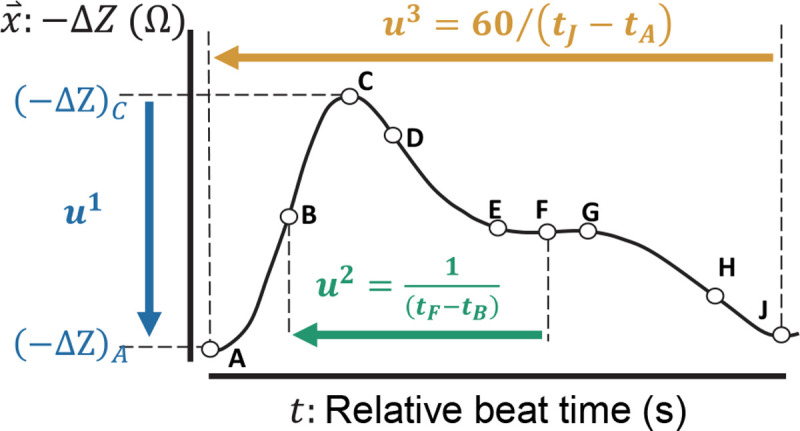
Bioimpedance beat feature definitions. The change in (inverted) bioimpedance signal amplitude, −Δ*Z*, is shown. −ΔZ increases with the arrival of the blood pressure pulse wave at the artery due to an increase in the artery volume. The amplitude change, *u*^1^, provides a proxy for the amount of arterial expansion. The second feature, *u*^2^, corresponds to the inverse of the relative time difference between the forward traveling (i.e., systolic) wave and reflection wave giving an estimate of the pulse wave velocity (PWV). The third feature, *u*^3^ gives beat-to-beat heart rate.

The feature definitions are shown in Figure 2. We share the details of bioimpedance signal pre-processing and the relationships regarding arterial dynamics in the Methods section and Supplementary Figure 2. For each blood pressure parameter (SBP, DBP, and PP), we trained a separate model with output prediction based on the type of blood pressure provided as ground truth data during supervised training.

### Blood pressure estimation

#### Study design.

We use three previously obtained bioimpedance datasets: (i) *graphene-HGCPT*^3^; (ii) *calfree-HGCPT*^30^; (iii) *ring-CPT*^31^. These datasets include bioimpedance waveforms captured with a wearable form-factor sensor placed along the participants’ wrist (i.e., radial) and finger (i.e., digital) arteries (see Supplementary Figure 1). For all datasets, the reference BP values come from a medical-grade BP monitoring finger cuff device, Finapres NOVA. The details of the datasets and human subject participation are shared in the Methods section. We build personalized models (i.e., models trained and tested on the data from the same participant) for *N* = 15 individuals (graphene-HGCPT dataset: *N* = 6, calfree-HGCPT dataset: *N* = 5, ring-CPT dataset: *N* = 4). All three datasets include wide BP ranges, reaching to hypertensive scale (i.e., systolic >140 mmHg, and diastolic >90 mmHg), due to the inclusion of BP elevation maneuvers (e.g., hand gripper: HG, cold-pressor test: CPT) in the standard protocol during data collection.

#### PINN model training, testing and performance evaluation.

**Figure 3.**
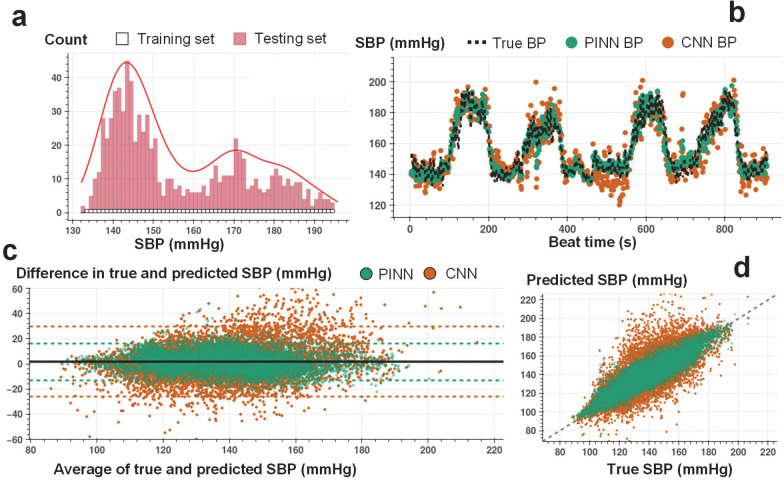
Beat-to-beat SBP estimation. **a.** Histogram for the training and testing set instances for varying SBP values used in PINN model training for a single individual (SID 15). For each 1 mmHg increase in SBP, we randomly select 1 data point for supervised model training, whereas all other points are used in model testing. **b.** Beat-to-beat SBP estimation (SID 15) based on PINN (shown in green) and reference conventional CNN (shown in orange) models trained with the same number of instances and corresponding true SBP (shown in dashed black). PINN shows a more precise fit to the reference SBP. **c.** Bland-Altman analysis with data analyzed for a total of N=15 subjects for PINN (green, ME: 1.3 mmHg, SD: 7.7 mmHg) and conventional CNN (orange, ME: 1.8 mmHg, SD: 14.4 mmHg) models. **d.** Pearson’s correlation analysis with data analyzed for a total of N=15 subjects for PINN (green, correlation coefficient r: 0.90) and conventional CNN (orange, correlation coefficient r: 0.73) models.

The proposed implementation of PINNs provides unique opportunities to train with minimal ground truth data for accurate time series translation. In contrast, traditional state-of-the-art ML and DNN models built for time series regression require training on large amounts of labeled data to offer acceptable performance. We assess the PINN performance against the CNN having the same neural network architecture for estimating SBP, DBP, and PP with minimally labeled data. The minimal training criterion is defined as neural network training with a set of labeled training instances, with each instance in the set randomly selected from the uniformly distributed and segmented BP values (see Figures 3a, 3b, and 3c, and Methods section for details of the train and test split criteria). The training labels covering different BP levels allow the PINNs to learn the Taylor polynomial approximating the complex input-output distribution across the complete dataset. Supplementary Tables 4–12 show the percent of training instances for each participant under the minimal training criterion.

**Figure 4.**
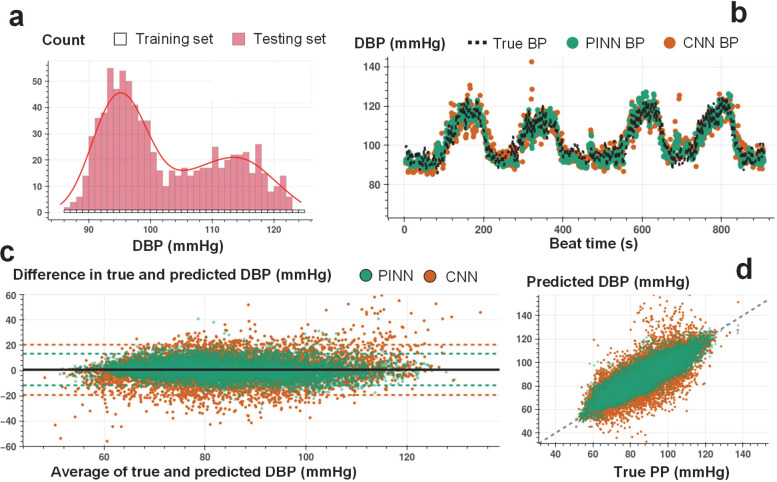
Beat-to-beat DBP estimation. **a.** Histogram for the training and testing set instances for varying DBP values used in PINN model training for a single individual (SID 15). For each 1 mmHg increase in DBP, we randomly select 1 data point for supervised model training, whereas all other points are used in model testing. **b.** Beat-to-beat DBP estimation (SID 15) based on PINN (shown in green) and reference conventional CNN (shown in orange) models trained with the same number of instances and corresponding true DBP (shown in dashed black). PINN shows a more precise fit to the reference DBP. **c.** Bland-Altman analysis with data analyzed for a total of N=15 subjects for PINN (green, ME: 0.6 mmHg, SD: 6.4 mmHg) and conventional CNN (orange, ME: 0.5 mmHg, SD: 10.1 mmHg) models. **d.** Pearson’s correlation analysis with data analyzed for a total of N=15 subjects for PINN (green, correlation coefficient r: 0.89) and conventional CNN (orange, correlation coefficient r: 0.77) models.

**Figure 5.**
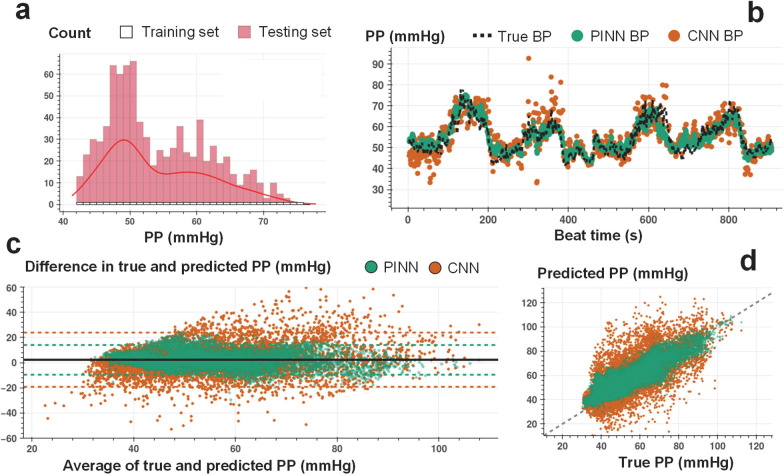
Beat-to-beat PP estimation. **a.** Histogram for the training and testing set instances for varying PP values used in PINN model training for a single individual (SID 15). For each 1 mmHg increase in SBP, we randomly select 1 data for supervised model training, whereas all other points are used in model testing. **b.** Beat-to-beat PP estimation (SID 15) based on PINN (shown in green) and reference conventional CNN (shown in orange) models trained with the same number of instances and corresponding true PP (shown in dashed black). PINN shows a more precise fit to the reference PP. **c.** Bland-Altman analysis with data analyzed for a total of N=15 subjects for PINN (green, ME: 2.2 mmHg, SD: 6.1 mmHg) and conventional CNN (orange, ME: 2.4 mmHg, SD: 10.9 mmHg) models. **d.** Pearson’s correlation analysis with data analyzed for a total of N=15 subjects for PINN (green, correlation coefficient r: 0.89) and conventional CNN (orange, correlation coefficient r: 0.72) models.

Figures 3b, 4b, and 5b show an example of beat-to-beat SBP, DBP, and PP estimations with PINN and conventional CNN trained with less than ~10% of the labeled data and tested on the remaining ~90% (Supplementary Figures 3–5 show PINN and CNN estimations of SBP, DBP, and PP for all participants). Under the same training constraint, we observe that PINNs show superior performance against conventionally trained CNNs in capturing localized changes in blood pressure yielding a higher correlation with lower absolute errors. Supplementary Tables 4–12 show PINN and CNN performances based on the mean error (ME), the standard deviation of the error (SDE), root-mean-squared error (RMSE), and Pearson’s correlation coefficient values in estimating SBP, DBP, and PP, respectively, for all participant from three datasets. PINNs, on average, improve the conventional model by 47%, 35%, and 39% for SBP, DBP, and PP, respectively (based on RMSE values averaged over all participants). Supplementary Tables 13–15 provide the results achieved with the PINNs presented based on the AAMI standard for BP devices^32^, where PINNs demonstrate a performance within the Grade A classification set by the standard, for all three datasets.

We conduct Bland-Altman and Pearson’s correlation analyses on the estimated vs. true SBP, DBP, and PP values for PINN and conventionally trained CNN models. The analyses results are shared in Figures 3c–d, 4c–d, and 5c–d, respectively (Bland-Altman, ME (SD) in mmHg, SBP, with PINN: 1.3 (7.6), with CNN: 1.8 (14.4); DBP, with PINN: 0.6 (6.4), with CNN: 0.5 (10.1); PP, with PINN: 2.2 (6.1), with CNN: 2.4 (10.9), Pearson’s analysis, correlation coefficient, r, SBP, with PINN: 0.90, with CNN: 0.73; DBP, with PINN: 0.89, with CNN: 0.77; PP, with PINN: 0.89, with CNN: 0.72). PINNs achieve, on average, 32%, 23%, and 69% percent higher correlation for SBP, DBP, and PP, respectively when compared to the conventional CNNs trained with the same amount of labeled data (per-subject results averaged over all participants, Pearson’s correlation r, SBP/DBP/PP, with PINN: 0.81/0.76/0.66, with CNN: 0.61/0.62/0.39).

We provide an additional comparison of the performance obtained with proposed PINNs with three state-of-the-art time series regression models: AdaBoost regressor^33^, Rocket regressor^34^, Random Forest regressor^35^. These models are trained with; (i) minimal training criterion, (ii) 4-fold cross-validation (~75% labeled data is used in training), (iii) 8-fold cross-validation (~88% labeled data is used in training). The results of this analysis are shared in Supplementary Tables 1–3, showing that compared to the state-of-the-art time series regression models PINNs retain low RMSE and high correlations with less amount of labeled data used in training (see Methods section for the details regarding the regression models). On average across all datasets, the RMSE and correlation for the above mentioned regression models range from 8.9–10.8 mmHg, 0.57–0.62 for SBP and from 6.6–8.1 mmHg, 0.57–0.64 for DBP, while PINN offers 7.1 mmHg and 0.82 for SBP and 5.7 mmHg and 0.76 for DBP, respectively.

#### Interpretation of Taylor polynomial parameters.

The neural network learning obtains function *f* that approximates the complex relationship between input and output. Due to the presence of a physics-based loss in the loss function, after network optimization, this function has a minimal difference with the evaluations of approximated Taylor polynomial, *P*. This polynomial is constructed with the use of features extracted from input times series measurements that are physiologically relevant to BP, e.g., *u*^1^ feature and peripheral blood volume change. Therefore, the parameters of *P* relate these features into complex cardiovascular parameters based on the underlying physiological mechanism. In addition, when this underlying physiological mechanism remains the same (e.g., different iterations of the cold-pressor test), these parameters are expected to demonstrate comparable behavior. However, if the input features have low correlation with the proxy hemodynamic parameter (e.g., caused by noise in measurements due to motion artifacts), the association between the parameters of *P* and the underlying physiological mechanism may become convoluted. Nevertheless, even in this case, PINNs retain high accuracies since the Taylor polynomials still provide a representation of non-trivial input-output mapping that guides the model predictions.

**Figure 6.**
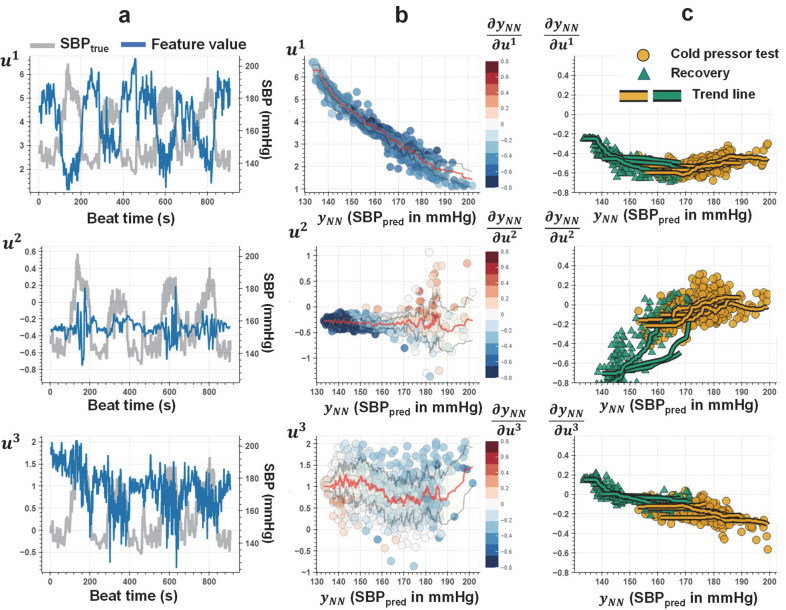
Parameters of the Taylor’s approximation of BP using physiological input features. The analysis results are obtained from the PINN model trained on a single individual’s SBP data (SID15). **a.** Beat-to-beat values for the bioimpedance input features (left y-axis), $\vec{u}:\left\{u^{1},u^{2},u^{3}\right\}$, and SBP (right y-axis) plotted against the time for each segmented beat. **b.** Scattered plots of the features, *u*^1^,*u*^2^, and *u*^3^ against the corresponding SBP predictions obtained with the neural network, *y*_*NN*_. Red and gray lines show the mean and ± standard deviation of the feature values for varying SBP. The colors show the values of the gradients for each point, i.e., *∂y*_*NN*_/*∂u*^1^, *∂y*_*NN*_/*∂u*^2^, and *∂y*_*NN*_/*∂u*^3^ calculated by auto-differentiation of PINN model output, *y*_*NN*_, with respect to each dimension of $\vec{u}$. For example, the consistent blue tones for the top plot refers to a consistent negative value for the gradient term *∂y*_*NN*_/*∂u*^1^, in agreement with the distribution of the *u*^1^ feature values showing a decrease for increasing *y*_*NN*_ predictions. **c.** Scattered plots of the gradient values *∂y*_*NN*_/*∂u*^1^, *∂y*_*NN*_/*∂u*^2^, and *∂y*_*NN*_/*∂u*^3^ for varying *y*_*NN*_. Colors represent the specific BP elevation maneuver for each data point, such that: yellow points correspond to the values obtained during cold-pressor test (CPT); green points correspond to the values obtained during the recovery session. The solid lines represent the trend line for each CPT or recovery session iteration, calculated from the average value for each integer SBP value. The observation for *u*^1^ feature values showing a strong negative correlation with SBP agrees with the negative value for the *∂y*_*NN*_/*∂u*^1^. The increase in magnitude for the gradient with increasing SBP can be associated with the volume-pressure dynamics driven by the arterial wall compliance. Subplots **a** and **b** for *u*^2^ show sudden changes for high SBP values and sustained changes for the remaining times. The gradient for this feature therefore shows amplified response for certain values of SBP, as shown in subplots **b** and **c**. The gradient for the third feature, i.e., rate of change in SBP with changing beat-to-beat heart rate, shows positive values for lower ranges of SBP and negative values for higher ranges of SBP. This change in polarity may be explained due to increased vagal tone dominating over the sympathetic activity with increasing SBP during the CPT.

For a proof-of-concept, we select a participant (SID15) whose first input feature shows high correlations with SBP (absolute value of Pearson’s correlation coefficient 0.9, see Figure 6a–b) and conducted a post-analysis of the PINN trained over minimally provided SBP labels (e.g. 65 out of 878 ground truth labels used for training). Partial differentials (i.e., gradients) calculated with auto-differentiation are defined for SBP predictions and input features, $\vec{u}$. We split the testing dataset into different subsets and trials representing different CPT and recovery sessions and plotted the partial differential value distribution across varying SBP. Through this analysis, we aim to assess; (i) the general behavior of the gradient-output distribution, (ii) the changes in this distribution for different sessions, and (iii) the consistency of this distribution across multiple iterations of the same type of session. Figure 6 shows the results of this analysis, plotted separately for all three features in $\vec{u}$.

The gradient for the first feature measures a relative change in blood pressure with a change in *u*^1^, which provides a proxy for arterial volume change in the peripheral arteries (e.g., digital, radial)^22^. The sympathetic stimulation caused by an external stressor (e.g., during CPT) leads to an increase in SBP^36,37^. Meanwhile, the peripheral arteries may observe vasoconstriction (i.e., narrowing of blood vessels by small muscles in their walls)^38,39^. Therefore, a negative gradient between BP and peripheral artery volume may be observed. We observe that *u*^1^ decreases with increasing *y*_*NN*_, where the corresponding gradient, *∂y*_*NN*_/*∂u*^1^ has a consistent negative amplitude in agreement with the feature behavior (Figure 6a–c top subplots). This negative amplitude can be associated with the vessel vasoconstriction due to CPT. The change in volume/pressure is also a factor of the arterial characteristics – e.g., artery compliance, that exhibit a non-linear pressure dependent relationship^2,40,41^. We observe the magnitude of the gradient increases with increasing SBP, which can be associated with the underlying arterial wall compliance characteristics that drive the volume-pressure relationships (See Methods section and Supplementary Figure 2).

The second-feature gradient (i.e., *∂y*_*NN*_/*∂u*^2^ ) is related to the non-linear relationship between PWV and SBP (See Methods and Supplementary Figure 2). This hemodynamic parameter is conventionally calculated based on measurements across proximal and distal points along the arterial tree ^42,43^. The physical separation of two measurement sites enables to capture of the time delay for the arrival of the BP pulse wave, i.e., pulse transit time (PTT), where PWV is *d*/*PTT*, with *d* being the distance between distal and proximal sensors. In our case, *u*^2^, only provides a proxy to PWV^28,29^, given that there is only a single-channel bioimpedance measurement used in extracting *u*^2^. We observe that this feature shows sudden changes in a short time frame for higher levels of SBP, dominating the changes at lower SBP levels. Therefore, the corresponding gradient result in having high magnitudes at lower SBP levels achieved during recovery sessions (green triangles in Figure 6c subplots), and values closer to zero at higher SBP levels achieved during CPT (yellow circles in Figure 6c subplots). Nevertheless, the decrease in magnitude with increasing SBP can be associated with the non-linear SBP-PWV relationship (see Methods and Supplementary Figure 2).

The third feature, *u*^3^, measures beat-to-beat HR, where its relationship with BP exhibits dynamic nature, affected by various physiological feedback mechanisms (e.g., vagal activity, baroreceptor reflex)^13,14^. It has been previously shown that during the CPT, HR may show an initial increase (e.g., increased sympathetic activity) followed by a decrease (e.g., increased vagal tone) with increasing SBP. We observe a similar pattern in the feature distribution against SBP as shown in Figure 6b (see mean trend line in red color). The gradient for the third feature, i.e., rate of change in SBP with changing beat-to-beat heart rate, shows positive amplitudes for lower ranges of SBP and negative amplitudes for higher ranges of SBP in agreement with the mean trend in the feature distribution. This change in polarity may be explained by the increased vagal tone dominating the sympathetic activity with increasing SBP during the CPT^13–15^. To further assess the consistency of the parameters establishing the Taylor polynomial across different amounts of information provided to the neural network, an additional analysis is provided. The purpose of this analysis is to demonstrate that the optimized PINN models obtain a consistent approximation of the input-output relationships defined by the Taylor polynomial under varying ground truth data used in model training. To assess this, we train a total of 137 separate models, with each model corresponding to a different number of training instances and data coming from a single individual (SID15): we create a set of initial training points, with each point randomly picked from BP data sorted and segmented into bins, where each bin has 0.5 mmHg steps. This results in 125, 77, and 69 training points for SBP, DBP, and PP, respectively. We start from a single training point and gradually increased the number of training points with two randomly picked points from the aforementioned training sets (e.g., this resulted in 63 models for SBP, labeled training instances: one to 125).

**Figure 7.**
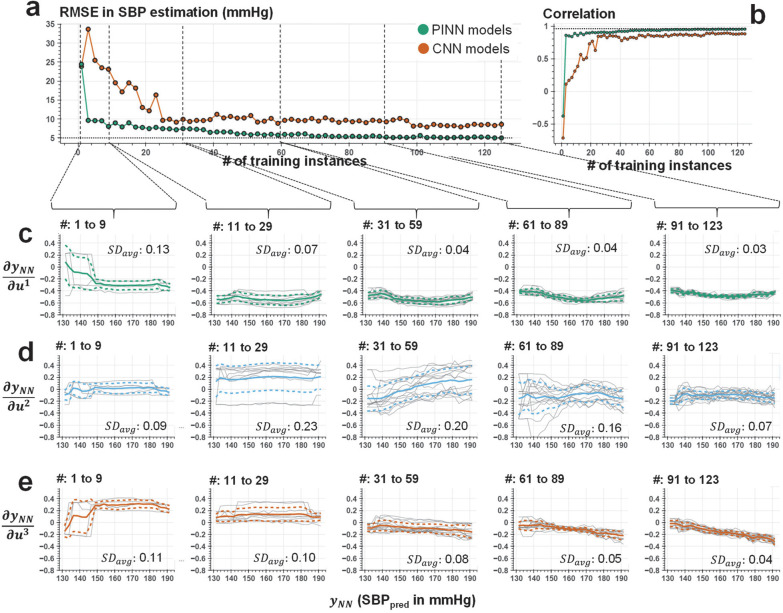
PINN model behavior under increasing number of training instances. **(a-b)** Root-mean-squared error (RMSE) and Pearson’s correlation coefficient in estimating SBP for PINN (green) and conventional CNN (orange) models trained over varying amounts of labeled instances. We observed that the PINN models retain consistently high performance (less than 10 mmHg RMSE and 0.85 correlation) when trained for more than ten labeled data points. **(c-e)** The distribution of the gradients, *∂y*_*NN*_/*∂u*^1^, *∂y*_*NN*_/*∂u*^2^, and *∂y*_*NN*_/*∂u*^3^, respectively, calculated as a part of the Taylor polynomial approximated for each model and grouped based on the number of labeled instances (see subplot titles) used in model training. Gray lines show the individual model trends, whereas solid and dashed colored lines represent the mean trend and standard deviation among all models for the given range of training instances, respectively. The subplot legends (i.e., *SD*_avg_) show standard deviation of the gradient values calculated across the group for each SBP and averaged across all points of SBP. A decrease in *SD*_avg_ is observed among the models trained over higher number of instances, in agreement with the obtained prediction performances. We observe that *∂y*_*NN*_/*∂u*^1^ and *∂y*_*NN*_/*∂u*^3^ show a consistent distribution (less than 0.10 standard deviation in the discovered trend calculated within the group) with as low as fewer than 40 labeled training instances. Whereas the second gradient, *∂y*_*NN*_/*∂u*^2^, demonstrates a higher deviation in constructed trend due to the complex behavior of the second feature, *u*^2^, that exhibit sudden changes during high levels of SBP (see Figure 6a–b middle subplots).

Figure 7a–b and Supplementary Figure 6a–b show the testing error for PINN and conventional CNN models based on RMSE and Pearson’s correlation coefficient, as the number of labeled training instances grows for SBP, DBP, and PP, respectively. We observe that the PINN models retain consistently high performance (less than 10 mmHg RMSE and 0.85 correlation) when trained for more than ten labeled data points. Figure 7c–e, show the distribution of the gradients, $\partial y_{NN}/\partial \vec{u}$, with respect to *y*_*NN*_, respectively, where the distributions are grouped based on the number of labeled instances used for training. We observe that *∂y*_*NN*_/*∂u*^1^ and *∂y*_*NN*_/*∂u*^3^ show a consistent distribution (less than 0.10 standard deviation in the discovered trend calculated within the group) with as low as fewer than 40 labeled training instances. Whereas the second gradient, *∂y*_*NN*_/*∂u*^2^, demonstrates a higher deviation in discovered trend due to the highly varying distribution of the second feature, *u*^2^, that exhibit sudden changes during high SBP values. The results of this analysis demonstrate the potential of using a limited number of discrete ground truth data points to train the neural networks that produce robust predictions for the remaining majority of input (e.g., more than N=800 samples) based on the unique integration of Taylor polynomial to the model training. Details of this analysis is shared in the Methods section.

## Discussions

Our novel contributions in this paper are summarized as follows: (i) we present a novel use of PINNs to extract actionable information from time series data for precision medicine with reduced dependency on ground truth information. Although the PINNs have already been introduced, conventionally they require well-defined generalized partial differential equations (PDEs). We propose to obtain PDEs at a personalized level by utilizing Taylor’s approximations for the gradually changing known cardiovascular relationships between input and output and integrating it into neural network training. This leads to robust predictions with training on minimal ground truth data. (ii) Our proposed technique adds interpretability to the network by establishing relationships with underlying cardiovascular dynamics. We show that the parameters of Taylor’s approximation remain consistent across iterations of varying BP elevation maneuvers (e.g., CPT) and show associations with known cardiovascular dynamics. (iii) We demonstrate the effectiveness of proposed PINNs through a comprehensive case study on continuous BP estimation with wearables. We test our models across different bioimpedance-BP datasets, including a total of N=15 participants, with various kinds of sensors (see Supplementary Figure 1) covering wide ranges of SBP, DBP, and PP. We show that PINN models retain high estimation accuracies for all individuals while decreasing the required amounts of ground truth data used in training, on average, by a factor of 15 (see Supplementary Tables 1–3). In addition, we compare the PINN performance against a neural network of the same architecture that is trained separately with the same amounts of labeled data, where PINNs obtain significantly higher performance (see Supplementary Tables 4–15 and Supplementary Figures 3–5).

The Taylor polynomial calculated with PINNs offers an approximation of the input-output dynamics based on the boundary conditions provided with limited ground truth data. The use of Taylor polynomial approximation over hand-crafted equations (e.g., linear regression models for PTT and BP^44^) for building PINNs address several important challenges, such as; (i) infeasibility of defining static equations that work under differing physiological contexts (e.g., exercise, stress, recovery, rest)^45^, (ii) inability to represent and mimic complex cardiovascular dynamics as it is challenging to estimate the underlying parameters for personalization.

## Methods

### Study description and human participation.

Three datasets are used in model training and evaluation: graphene-HGCPT^3^, calfree-HGCPT^30^, ring-CPT^31^. Each dataset contains the raw time series measurements obtained with a wearable-form factor bioimpedance sensor, and the corresponding reference BP values acquired from a medical-grade finger cuff (Finapres NOVA). The Graphene-HGCPT dataset involves N=6 participants (1/5 female/male, age range/median: 21–31/25) that were asked to go through multiple sessions of a BP elevation routine involving HG exercise followed directly by CPT and recovery. The participants wore bioimpedance sensors that used graphene e-tattoos placed at the participants’ wrists along the radial artery. The calfree-HGCPT dataset involves N=5 participants (all male, age range/median: 20–25/23) that were asked to go through multiple sessions of the HG and CPT protocols. The participants wore a silver electrode-based wristband at different positions. We use data collected at POS1 corresponding to the placement of electrodes aligned with the participants’ radial arteries. Ring-CPT dataset involves N=4 participants (all male, age: range/median 19–26/21) that were asked to go through multiple sessions of CPT and recovery. The bioimpedance data was collected with a ring-worn bioimpedance sensor placed at the participants’ ring fingers.

### Bioimpedance signal pre-processing and feature extraction.

Bioimpedance modality measures deep tissue characteristics of the human body: tissue and cell compositions and their transient behavior due to physio-mechanical activities (e.g., blood flow, respiration, body fluid shifts, body fat-muscle composition changes) based on a very small, high-frequency non-invasive electrical signal injected between two contact points. The induced voltage signal obtained at additional pairs of contacts changes with the tissue composition changes and their electrical properties. When bioimpedance sensors are placed along the arteries, the acquired signal changes quasi-periodically with the change in the artery volume due to the arrival of the BP pulse wave at each heartbeat cycle. We use simultaneously acquired and synchronized bioimpedance and blood pressure (Finapres NOVA) data stream in each dataset. We segmented the data into beat-to-beat intervals. For the ring-CPT and calfree-HGCPT datasets, we use the delta bioimpedance waveform (i.e., Δ*Z*: change in bioimpedance in the order of 50–100 mΩ, through the removal of baseline impedance – *Z*_0_ – in the order of 20–100 Ω.) normalized by *Z*_0_, and ΔZ with no normalization of the graphene-HGCPT dataset due to the unavailability of *Z*_0_ baseline value.

We extract nine fiducial points from each beat-to-beat bioimpedance waveform as shown in Figure 2 to calculate physiological features, *x*^(1)^, *x*^(2)^, and *x*^(3)^, where *x*^(1)^ = (ΔZ)_*A*_ – (ΔZ)_*C*_, *x*^(2)^ = 1/(*t*_*F*_ – *t*_*B*_), and *x*^(3)^ is 60/(*t*_*J*_ – *t*_*A*_), i.e. heart rate, with (Δ*Z*)_*i*_ and *t*_*i*_ being the delta bioimpedance amplitude and time instance of the *i*-th fiducial point (i.e., *i* ∈ {*A*, *B*, …, *J*}). We apply a three-beat moving average with one-beat overlap to the segmented bioimpedance waveforms and calculated physiological features and blood pressure values (i.e., systolic, diastolic, and pulse pressure). Prior to training of the model, we normalize (zero mean and unity standard deviation) all model inputs and outputs based on the complete dataset. Prior to the statistical analyses, the model output values are converted back to mmHg units based on the initial mean and standard deviation values used in normalization.

### Neural network design, hyperparameter selections, and model training.

We use identical model architecture, input-output structure, and layer hyperparameters for PINN and conventional CNN models for fair analysis. We define two separate model inputs, $\vec{x}$ and $\vec{u}$, representing the zero-padded, down-sampled (sampling rate of 30 Hz) and segmented bioimpedance beats, and the features (i.e., three physiological features extracted from bioimpedance), respectively. The segmented bioimpedance beat, $\vec{x}$, is connected to a two-layer 1D-CNN network (first layer number of filters: 32, kernel size: 5, activation: ‘RELU’, second layer number of filters: 64, kernel size: 3, activation: ‘RELU’), with a max-pooling (pool size: 3, strides: 1) is applied to its output, followed by flattening. We then use a concatenation layer to combine flattened CNN outputs with $\vec{u}$. The concatenated layer was then connected to a series of fully connected network layers (layer-1 number of neurons: 60, activation: ‘RELU’; layer-2 number of neurons: 1) providing the model estimations.

### Model training with minimally labeled data.

We designed a unique criterion to train neural network models with minimum labeled data and tested them over the complete BP range. To achieve this, we divided each participant’s data into *K* segments, with *K* being the output BP range (i.e., corresponding to 1 mmHg increment between each consecutive segment), and randomly selected one output label from each segment to be included in the supervised training set. For example, in a dataset with 1000 samples and ranging systolic pressure of 120–160 mmHg (*K* = 40), the train set includes only 40 samples (4% of the dataset for supervised training), while the test set includes 960 samples (96% of the dataset used for testing). The PINNs and conventional CNNs were trained with minimal labeled data. To provide a fair comparison between the two models, we terminated the model training when the supervised training losses reached 0.01. Additionally, we observe that beyond a certain point, the conventional neural networks tend to overfit the training data, further increasing the testing error, while the PINN model is prevented from overfitting due to the physics loss in the objective function.

### Train and test of BP estimation with state-of-the-art time series regression models.

We tested three time series regression models: AdaBoost regressor^33^, Rocket regressor^34^, Random Forest regressor^35^. The models are retrieved from publicly available Python (version 3.9) libraries: for AdaBoost regressor, *scikit-learn* (https://scikit-learn.org/)^46^, for Rocket and Random Forest regressors, *sktime* (https://github.com/sktime/sktime/)^47^. To run AdaBoost and Random Forest regressors, we extracted in total 16 features from inverted bioimpedance waveforms (eight out of 16: time-based, remainder eight out of 16: amplitude-based), based on a total of nine fiducial points extracted from the bioimpedance waveform (see Figure 2). AdaBoost regressors used ensemble of Decision Tree regressors with maximum depth of 15, and number of estimators of 100. Random Forest regressors used an ensemble of Decision Tree regressors built on random intervals, with minimum interval width of three, and number of estimators of 100. For Rocket regressor, the raw segmented bioimpedance waveforms were provided as inputs with the number of kernels selected as 100. For each model, three training criterion were tested: (i) minimal training criterion, (ii) 4-fold cross-validation, (iii) 8-fold cross validation. For K-fold cross-validation analyses, the dataset is divided into K-sets of equal length, where K-1 sets are used for training, and the remaining set is used for testing. In addition, to achieve fair comparison, we included the training instances resultant from minimal training criterion, as described earlier, in the training sets defined based on K-fold cross-validation.

### Model training with varying numbers of labeled training instances.

To compare model performance under a growing number of labeled training instances, we created an initial training set. This set was generated based on the minimal training criterion where the labeled data is split into *k* bins with *k* = *BP*_*range*_ × 2, where *BP*_*range*_ is the difference between the maximum and minimum BP values calculated separately for SBP, DBP, and PP. This divides the dataset into different bins with bin widths equaling 0.5 mmHg. We then randomly select one point from each bin resulting in *k* total of labeled data points for the initial training set. We train *k*/2 different models for each BP output for PINN and CNN, where each model received varying numbers of labeled training points, (an increment of two in the number of labeled training points for consecutive models). For example, the first model is trained with one labeled training instance, the second model is trained with three, and the *N*-th model is trained with 1 + *N* × 2 labeled training instances. For each model, we measure the performance against the reference BP using the test set corresponding to the ground truth BP values that are not included in the training.

### Hemodynamic relationships.

Systolic and diastolic blood pressure values correspond to the maximum and minimum pressure points in the artery. During systole, the heart ejects blood into the aorta, which then travels through the arterial tree. Pulse pressure is the difference between systolic and diastolic blood pressure. The changes in PP and SBP are proportional to volumetric changes based on the arterial wall characteristics defined by compliance^48^, where the parameters for the equation changes per individual (see Supplementary Figure 2).

The blood pressure pulse wave velocity (PWV) is also related to the arterial wall characteristics and its response to changing pressure. The relationship for PWV is defined as: $PWV=\sqrt{Eh/D\rho }$,^49^ where *D* is the diameter of the radial artery, *h* is the wall thickness of the radial artery, *ρ* is the average density of human blood, and *E* is the elastic modulus of the artery wall that has a positive correlation with blood pressure^50^.

HR and BP do not necessarily increase at the same rate due to different underlying CV control mechanisms. The relationship between HR and PP during CPT is shown to have varying correlations^13,14^. For certain individuals, reciprocal changes in cardiac autonomic regulation induce a sustained increase in HR with an increase in BP, while for others, CPT induces a decrease in HR after an initial increase, likely due to the co-activation of the vagal and sympathetic outflow at the heart level (see Supplementary Figure 2d).

### Performance metrics.

To assess the trained model performance on a test dataset, we calculate per-subject and group mean error (ME), the standard deviation of the error (SDE), root-mean-squared error (RMSE), along with confidence intervals and Pearson’s correlation coefficients, based on true and estimated blood pressure values. Additionally, we report the results according to the AAMI standard for BP devices^32^.

## Supplementary Material

Supplement 1

## Data Availability

The graphene-HGCPT dataset analyzed during the current study is available in the PhysioNet repository, doi.org/10.13026/ce62-pc98. The calfree-HGCPT and ring-CPT datasets analyzed during the current study are available from the corresponding author on reasonable request.
