# Supplementary material for: Physics-Informed Neural Networks for Modeling Physiological Time Series: A Case Study with Continuous Blood Pressure
Source: Res Sq. 2023 Jan 16:rs.3.rs-2423200. Preprint. [Version 1] doi: 10.21203/rs.3.rs-2423200/v1 (PMC9882661; doi:10.21203/rs.3.rs-2423200/v1)
Supplement: Supplement 1 [file NIHPPrs2423200v1-supplement-1.pdf]

Supplementary Information

Supplementary Tables

Supplementary Table 1. Graphene-HGCPT dataset. Model performances in SBP and DBP estimation. Values are averaged over all participants for each dataset.

BP estimation model	Training set criterion	% of training labels	Systolic BP		Diastolic BP	
			RMSE, mmHg	Correlation	RMSE,	Correlation
AdaBoost Regressor	Minimal training	7% for SBP,<br>5% for DBP	13.0	0.55	9.9	0.56
	4-fold cross-validation	~75%	11.0	0.53	8.9	0.48
	8-fold cross-validation	~88%	10.8	0.54	8.7	0.52
Rocket Regressor	Minimal training	7% for SBP,<br>5% for DBP	12.4	0.57	9.6	0.54
	4-fold cross-validation	~75%	10.9	0.55	9.0	0.46
	8-fold cross-validation	~88%	10.5	0.58	8.6	0.5
Random Forest Regressor	minimal training	7% for SBP,<br>5% for DBP	11.6	0.58	8.9	0.59
	4-fold cross-validation	~75%	10.8	0.52	8.6	0.49
	8-fold cross-validation	~88%	10.4	0.56	8.3	0.52
CNN<br>(i.e., without $\mathcal{L}_{physics}$)	minimal training	7% for SBP,<br>5% for DBP	14.7	0.57	12.0	0.52
**PINN<br>(i.e. with $\mathcal{L}_{physics}$)**	**minimal training**	**7% for SBP,<br>5% for DBP**	**7.8**	**0.81**	**7.1**	**0.73**

Supplementary Table 2. Calfree-HGCPT dataset. Model performances in SBP and DBP estimation. Values are averaged over all participants for each dataset.

**BP estimation model**	**Training set criterion**	**% of training labels**	**Systolic BP**		**Diastolic BP**	
			**RMSE, mmHg**	**Correlation**	**RMSE,**	**Correlation**
AdaBoost Regressor	Minimal training	5% for SBP, 4% for DBP	11.3	0.58	8.5	0.62
	4-fold cross-validation	~75%	10.5	0.55	7.0	0.65
	8-fold cross-validation	~88%	9.9	0.58	6.8	0.66
Rocket Regressor	Minimal training	5% for SBP, 4% for DBP	11.5	0.53	8.7	0.56
	4-fold cross-validation	~75%	11.0	0.47	7.7	0.60
	8-fold cross-validation	~88%	10.6	0.49	7.4	0.63
Random Forest Regressor	minimal training	5% for SBP, 4% for DBP	11.4	0.54	8.2	0.61
	4-fold cross-validation	~75%	10.4	0.52	7.3	0.61
	8-fold cross-validation	~88%	10.0	0.56	6.9	0.65
CNN (i.e., without $\mathcal{L}_{physics}$)	minimal training	5% for SBP, 4% for DBP	13.9	0.53	8.7	0.65
**PINN (i.e. with $\mathcal{L}_{physics}$)**	**minimal training**	**5% for SBP, 4% for DBP**	**8.1**	**0.77**	**6.1**	**0.76**

Supplementary Table 3. Ring-CPT dataset. Model performances in SBP and DBP estimation. Values are averaged over all participants for each dataset.

BP estimation model	Training set criterion	% of training labels	Systolic BP		Diastolic BP	
			RMSE, mmHg	Correlation	RMSE,	Correlation
AdaBoost Regressor	Minimal training	12% for SBP,<br>7% for DBP	7.2	0.74	5.3	0.69
	4-fold cross-validation	~75%	6.8	0.72	4.9	0.64
	8-fold cross-validation	~88%	6.6	0.75	4.6	0.69
Rocket Regressor	Minimal training	12% for SBP,<br>7% for DBP	8.6	0.7	6.0	0.64
	4-fold cross-validation	~75%	7.8	0.69	5.0	0.65
	8-fold cross-validation	~88%	7.5	0.73	4.8	0.71
Random Forest Regressor	minimal training	12% for SBP,<br>7% for DBP	6.7	0.75	4.8	0.71
	4-fold cross-validation	~75%	6.7	0.72	4.8	0.64
	8-fold cross-validation	~88%	6.3	0.75	4.5	0.7
CNN<br>(i.e., without $\mathcal{L}_{physics}$)	minimal training	12% for SBP,<br>7% for DBP	8.6	0.76	5.2	0.72
**PINN<br>(i.e. with $\mathcal{L}_{physics}$)**	**minimal training**	**12% for SBP,<br>7% for DBP**	**5.4**	**0.88**	**3.8**	**0.80**

Supplementary Table 4. SBP estimation results for each participant in the graphene-CPT dataset. RMSE: root-mean-squared error, ME: mean error, SDE: standard deviation of the error, R: Pearson's correlation coefficient.

**SBP**	**Graphene-CPT**		**PINN**				**CNN**			
**Subject ID**	Percent of training instances	Total number of samples	RMSE (mmHg)	ME (mmHg)	SDE (mmHg)	R	RMSE (mmHg)	ME (mmHg)	SDE (mmHg)	R
**1**	3.67	1,878	6.98	0.48	6.97	0.84	10.76	-1.04	10.71	0.73
**2**	3.64	1,867	8.05	2.71	7.58	0.73	15.29	3.09	14.98	0.51
**3**	7.93	920	8.24	3.22	7.59	0.86	11.70	-0.73	11.68	0.77
**4**	8.25	727	7.32	1.17	7.22	0.84	10.92	1.68	10.79	0.70
**5**	8.35	599	8.32	3.96	7.32	0.69	13.23	4.6	12.41	0.32
**6**	7.83	1,060	8.04	0.6	8.02	0.88	26.29	7.62	25.16	0.37
**Average**	6.61	1,175	7.82	2.02	7.45	0.81	14.70	2.54	14.29	0.57

Supplementary Table 5. SBP estimation results for each participant in the calfree-HGCPT dataset. RMSE: root-mean-squared error, ME: mean error, SDE: standard deviation of the error, R: Pearson's correlation coefficient.

**SBP**	**Calfree-HGCPT**		**PINN**				**CNN**			
**Subject ID**	Percent of training instances	Total number of samples	RMSE (mmHg)	ME (mmHg)	SDE (mmHg)	R	RMSE (mmHg)	ME (mmHg)	SDE (mmHg)	R
**7**	4.83	1,574	8.22	2.98	7.66	0.82	18.30	1.06	18.27	0.40
**8**	3.52	1,278	7.39	2.27	7.03	0.59	10.77	2.77	10.41	0.39
**9**	5.37	1,453	8.73	0.8	8.70	0.80	16.12	2.91	15.85	0.53
**10**	6.47	1,237	8.49	1.58	8.34	0.85	12.98	2.6	12.72	0.74
**11**	7.12	955	7.45	0.63	7.42	0.78	11.29	2.1	11.09	0.60
**Average**	5.46	1,299	8.06	1.65	7.83	0.77	13.89	2.29	13.67	0.53

Supplementary Table 6. SBP estimation results for each participant in the ring-CPT dataset. RMSE: root-mean-squared error, ME: mean error, SDE: standard deviation of the error, R: Pearson's correlation coefficient.

**SBP**	**Ring-CPT**		**PINN**				**CNN**			
**Subject ID**	Percent of training instances	Total number of samples	RMSE (mmHg)	ME (mmHg)	SDE (mmHg)	R	RMSE (mmHg)	ME (mmHg)	SDE (mm Hg)	R
**12**	8.32	469	5.17	0.2	5.16	0.83	8.66	0.2	8.66	0.69
**13**	7.41	607	5.08	-0.89	5.00	0.82	7.91	-1.23	7.81	0.64
**14**	24.08	245	5.88	0.1	5.88	0.92	9.34	1.22	9.26	0.80
**15**	7.40	878	5.48	-0.32	5.47	0.95	8.50	-0.28	8.50	0.89
**Average**	11.80	550	5.40	-0.23	5.38	0.88	8.60	-0.02	8.56	0.76

Supplementary Table 7. DBP estimation results for each participant in the graphene-CPT dataset. RMSE: root-mean-squared error, ME: mean error, SDE: standard deviation of the error, R: Pearson's correlation coefficient.

DBP	Graphene-CPT		PINN				CNN			
**Subject ID**	Percent of training instances	Total number of samples	RMSE (mmHg)	ME (mmHg)	SDE (mmHg)	R	RMSE (mmHg)	ME (mmHg)	SDE (mmHg)	R
**1**	2.77	1,878	6.64	-0.81	6.59	0.77	8.79	-1.44	8.67	0.67
**2**	3.05	1,867	7.62	1.83	7.40	0.65	13.55	-1.39	13.48	0.46
**3**	4.78	920	6.51	2.47	6.03	0.72	9.75	3.53	9.09	0.61
**4**	7.70	727	6.52	0.06	6.52	0.85	9.61	1.8	9.44	0.71
**5**	7.68	599	6.38	1.23	6.26	0.77	12.59	-1.06	12.55	0.40
**6**	6.13	1,060	9.01	-0.35	9.00	0.63	17.69	2.64	17.49	0.27
**Average**	5.35	1,175	7.11	0.74	6.97	0.73	12.00	0.68	11.79	0.52

Supplementary Table 8. DBP estimation results for each participant in the calfree-HGCPT dataset. RMSE: root-mean-squared error, ME: mean error, SDE: standard deviation of the error, R: Pearson's correlation coefficient.

DBP	Calfree-HGCPT		PINN				CNN			
**Subject ID**	Percent of training instances	Total number of samples	RMSE (mmHg)	ME (mmHg)	SDE (mmHg)	R	RMSE (mmHg)	ME (mmHg)	SDE (mmHg)	R
**7**	3.11	1,574	5.82	1.11	5.71	0.82	8.20	1.2	8.11	0.71
**8**	3.05	1,278	5.93	2.88	5.18	0.60	8.04	3.07	7.43	0.51
**9**	3.44	1,453	7.01	1.83	6.77	0.80	8.44	0.92	8.39	0.74
**10**	5.74	1,237	6.17	-0.2	6.16	0.91	10.77	-1.47	10.67	0.77
**11**	4.29	955	5.62	1.07	5.52	0.66	7.87	1.32	7.76	0.51
**Average**	3.93	1,299	6.11	1.34	5.87	0.76	8.66	1.01	8.47	0.65

Supplementary Table 9. DBP estimation results for each participant in the ring-CPT dataset. RMSE: root-mean-squared error, ME: mean error, SDE: standard deviation of the error, R: Pearson's correlation coefficient.

DBP	Ring-CPT		PINN				CNN			
**Subject ID**	Percent of training instances	Total number of samples	RMSE (mmHg)	ME (mmHg)	SDE (mmHg)	R	RMSE (mmHg)	ME (mmHg)	SDE (mmHg)	R
**12**	6.18	469	4.54	0.47	4.51	0.63	6.71	-0.12	6.71	0.45
**13**	4.12	607	3.31	-0.03	3.31	0.73	3.73	0.65	3.68	0.73
**14**	14.29	245	3.31	-0.67	3.24	0.92	4.84	-1.18	4.70	0.84
**15**	4.67	878	4.08	-0.84	3.99	0.92	5.40	-0.26	5.39	0.85
**Average**	7.32	550	3.81	-0.27	3.76	0.80	5.17	-0.23	5.12	0.72

Supplementary Table 10. PP estimation results for each participant in the graphene-CPT dataset. RMSE: root-mean-squared error, ME: mean error, SDE: standard deviation of the error, R: Pearson's correlation coefficient.

**PP**	**Graphene-CPT**		**PINN**				**CNN**			
**Subject ID**	Percent of training instances	Total number of samples	RMSE (mmHg)	ME (mmHg)	SDE (mmHg)	R	RMSE (mmHg)	ME (mmHg)	SDE (mmHg)	R
**1**	1.54	1,878	3.65	1.53	3.31	0.74	6.53	0.18	6.53	0.33
**2**	2.57	1,867	10.56	5.99	8.70	0.39	19.63	7.34	18.21	0.10
**3**	6.20	920	6.11	2.99	5.33	0.86	9.74	2.2	9.49	0.70
**4**	3.85	727	4.58	-1.31	4.39	0.59	8.14	-2.88	7.61	0.20
**5**	5.84	599	7.69	3.1	7.04	0.61	16.44	-0.75	16.43	0.04
**6**	3.87	1,060	6.64	2.05	6.31	0.65	10.16	2.27	9.91	0.40
**Average**	3.98	1,175	6.54	2.39	5.85	0.64	11.77	1.39	11.36	0.30

Supplementary Table 11. PP estimation results for each participant in the calfree-HGCPT dataset. RMSE: root-mean-squared error, ME: mean error, SDE: standard deviation of the error, R: Pearson's correlation coefficient.

**PP**	**Calfree-HGCPT**		**PINN**				**CNN**			
**Subject ID**	Percent of training instances	Total number of samples	RMSE (mmHg)	ME (mmHg)	SDE (mmHg)	R	RMSE (mmHg)	ME (mmHg)	SDE (mmHg)	R
**7**	3.18	1,574	7.78	2.97	7.19	0.72	16.58	4.19	16.04	0.23
**8**	1.80	1,278	4.48	2.04	3.99	0.43	5.65	2.15	5.23	0.23
**9**	2.55	1,453	5.97	0.97	5.89	0.45	7.59	2.14	7.28	0.39
**10**	2.51	1,237	4.87	2.23	4.33	0.60	6.18	1.35	6.03	0.33
**11**	4.71	955	6.62	1.84	6.36	0.68	11.35	4.46	10.44	0.29
**Average**	2.95	1,299	5.94	2.01	5.55	0.58	9.47	2.86	9.00	0.29

Supplementary Table 12. PP estimation results for each participant in the ring-CPT dataset. RMSE: root-mean-squared error, ME: mean error, SDE: standard deviation of the error, R: Pearson's correlation coefficient.

**PP**	**Ring-CPT**		**PINN**				**CNN**			
**Subject ID**	Percent of training instances	Total number of samples	RMSE (mmHg)	ME (mmHg)	SDE (mmHg)	R	RMSE (mmHg)	ME (mmHg)	SDE (mmHg)	R
**12**	4.69	469	3.21	-0.48	3.17	0.81	4.30	0.01	4.30	0.71
**13**	3.62	607	2.95	0.31	2.94	0.75	3.51	0.8	3.42	0.71
**14**	16.33	245	7.72	2.68	7.24	0.66	9.34	4.4	8.23	0.48
**15**	4.10	878	3.52	0.34	3.50	0.89	5.62	0.87	5.55	0.77
**Average**	7.18	550	4.35	0.71	4.21	0.78	5.69	1.52	5.38	0.67

Supplementary Table 13. AAMI results for SBP estimation. ME: mean error, SDE: standard deviation of error.

	**PINN**		**CNN**	
**SBP**	ME (mmHg)	SDE (mmHg)	ME (mmHg)	SDE (mmHg)
AAMI standard	< 5	< 8	< 5	< 8
**Dataset**	ME (mmHg)	SDE (mmHg)	ME (mmHg)	SDE (mmHg)
Calfree-HGCPT	1.75	7.94	2.26	14.42
Graphene-CPT	1.81	7.54	2.15	15.27
Ring-CPT	-0.33	5.33	-0.30	8.45

Supplementary Table 14. AAMI results for DBP estimation. ME: mean error, SDE: standard deviation of error.

	**PINN**		**CNN**	
**DBP**	ME (mmHg)	SDE (mmHg)	ME (mmHg)	SDE (mmHg)
AAMI standard	< 5	< 8	< 5	< 8
**Dataset**	ME (mmHg)	SDE (mmHg)	ME (mmHg)	SDE (mmHg)
Calfree-HGCPT	1.37	6.01	1.03	8.65
Graphene-CPT	0.65	7.23	0.18	12.29
Ring-CPT	-0.32	3.90	-0.07	5.26

Supplementary Table 15. AAMI results for PP estimation. ME: mean error, SDE: standard deviation of error.

	**PINN**		**CNN**	
**PP**	ME (mmHg)	SDE (mmHg)	ME (mmHg)	SDE (mmHg)
AAMI standard	< 5	< 8	< 5	< 8
**Dataset**	ME (mmHg)	SDE (mmHg)	ME (mmHg)	SDE (mmHg)
Calfree-HGCPT	2.03	5.77	2.82	10.18
Graphene-CPT	2.82	6.61	2.30	12.80
Ring-CPT	0.38	3.93	1.01	5.27

Supplementary Figures

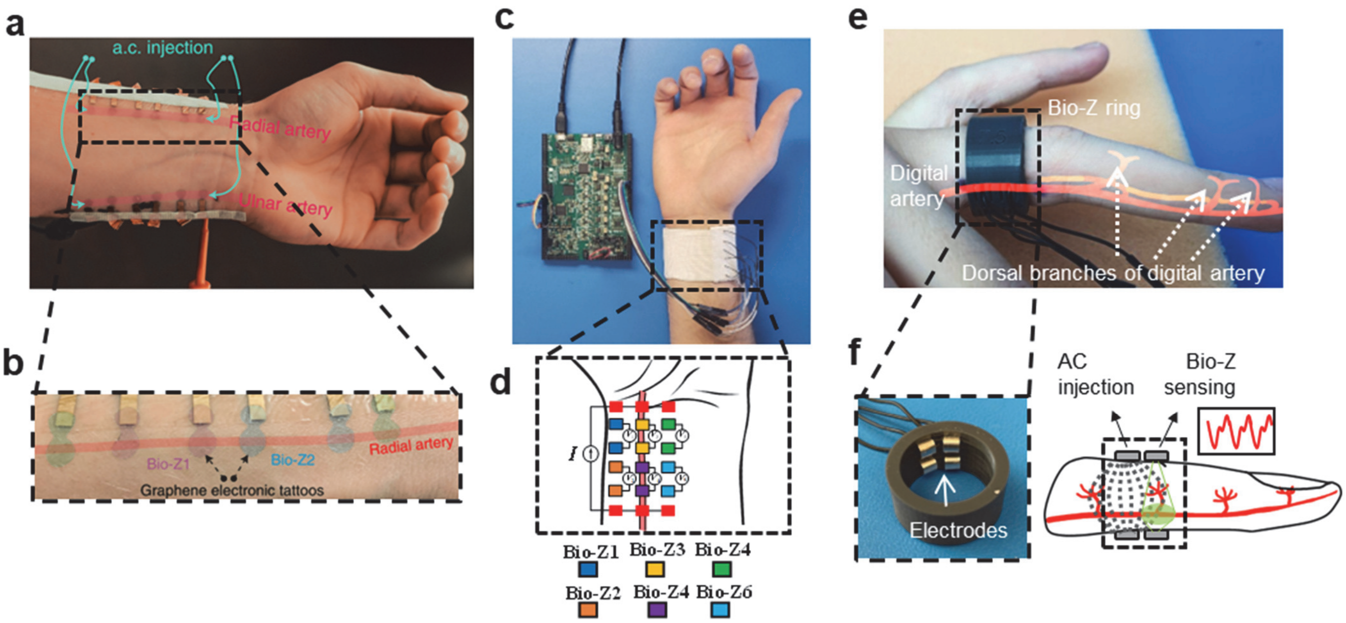

Supplementary Figure 1. Sensors used for bioimpedance signal acquisition. The bioimpedance sensing requires the injection of a high-frequency electric signal via a pair of electrodes building electrical contact with the skin, where a separate pair of electrodes are used to obtain the bioimpedance signal induced by this injected signal. The bioimpedance signal provides measurements of deep tissue characteristics of the human body: tissue and cell compositions and their transient behavior due to physio-mechanical activities (e.g., blood flow, respiration, body fluid shifts, body fat-muscle composition changes). When the bioimpedance sensors are placed along the arteries (e.g., radial artery on wrist, digital artery on finger), the acquired signal changes quasi-periodically with the arterial volume changes due to the arrival of the pulse wave at each heartbeat cycle. We used three bioimpedance datasets, where each dataset includes measurements obtained via bioimpedance sensors consisting of unique electrode types, capturing blood volumetric changes at participants' wrists or fingers. **(a-b)** In the graphene-HGCPT dataset, the bioimpedance sensor electrodes are based on atomically thin, self-adhesive, lightweight and unobtrusive graphene electronic tattoos (GETs)[3]. A total of 12 GETs with a surface area of 25 mm^2 are placed at the participants' wrists aligned with radial and ulnar arteries. In this work, we used bioimpedance signal obtained with electrodes marked as Bio-Z2 (blue color in subplot b). Reproduced with permission[3]. Copyright © 2022, Springer Nature. **(c-d)** In the calfree-HGCPT dataset, a wrist-worn array band consisting of 6 x 8 silver electrodes are used for bioimpedance signal acquisition[30]. Each electrodes sized 5 mm x 5 mm, with a 3.2 mm spacing between the electrodes. In this work, we used bioimpedance signal obtained with electrodes marked as Bio-Z3 (yellow color in subplot d). Reproduced with permission[30]. Copyright © 2022, Springer Nature. **(e-f)** In the ring-CPT dataset, a ring form-factor bioimpedance sensor consisting of 2 x 2 silver electrodes are used. The rings are easy to wear, not burdensome and provide ideal sensor-to-skin contact. Each participant wore a bioimpedance ring sensor on their ring fingers, where the sensing electrode pair within the sensor is aligned with the underlying digital artery.

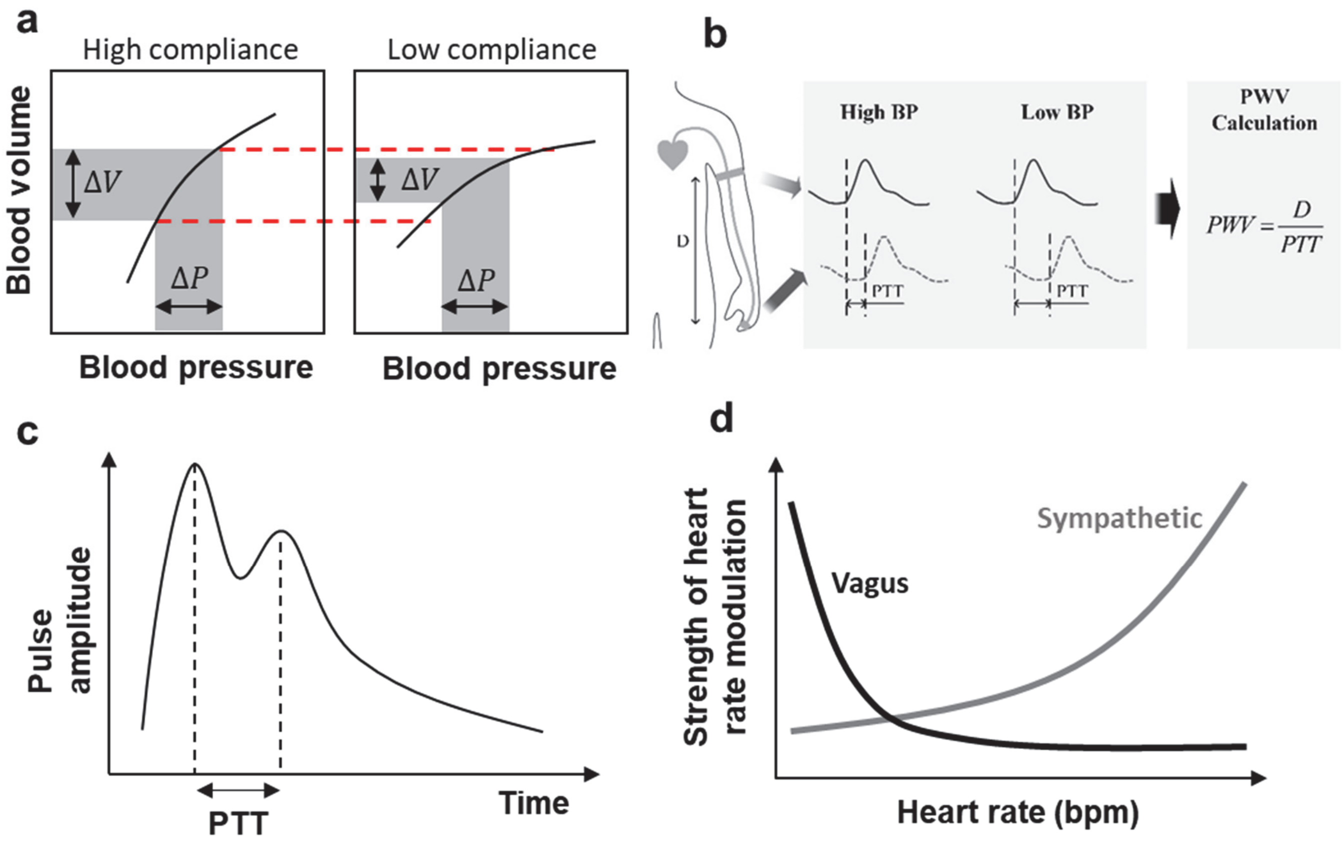

Supplementary Figure 2. Hemodynamic relationships. a. Blood volume – pressure relationship based on different arterial wall characteristics (i.e., varying compliance). An expansion in arterial diameter caused by blood arrival causes an increase in pressure exerted to the arterial wall. The change of pressure w.r.t. volume is dependent on the level of artery compliance[51]. **b.** Pulse wave velocity (PWV), pulse transit time (PTT) and BP relationship. Higher BP corresponds to a higher PWV and decreased PTT. Conventionally, PTT is measured using two (i.e. distal and proximal) points on the arterial tree[42,43]. Reproduced with permission[52]. Copyright © 2022, MDPI. **c.** Pulse wave analysis from wearable sensor measurements to capture a proxy for the PTT-PWV, based on the time difference between the arrivals of the first pressure wave and the reflected pressure wave to the sensing site[29]. **d.** Heart rate response to vagal nerve (e.g., vagal outflow) vs. sympathetic nervous system activities, which causes a variation in the heart rate response to a stressor (e.g., cold-pressor test) that increases blood pressure[13–15].

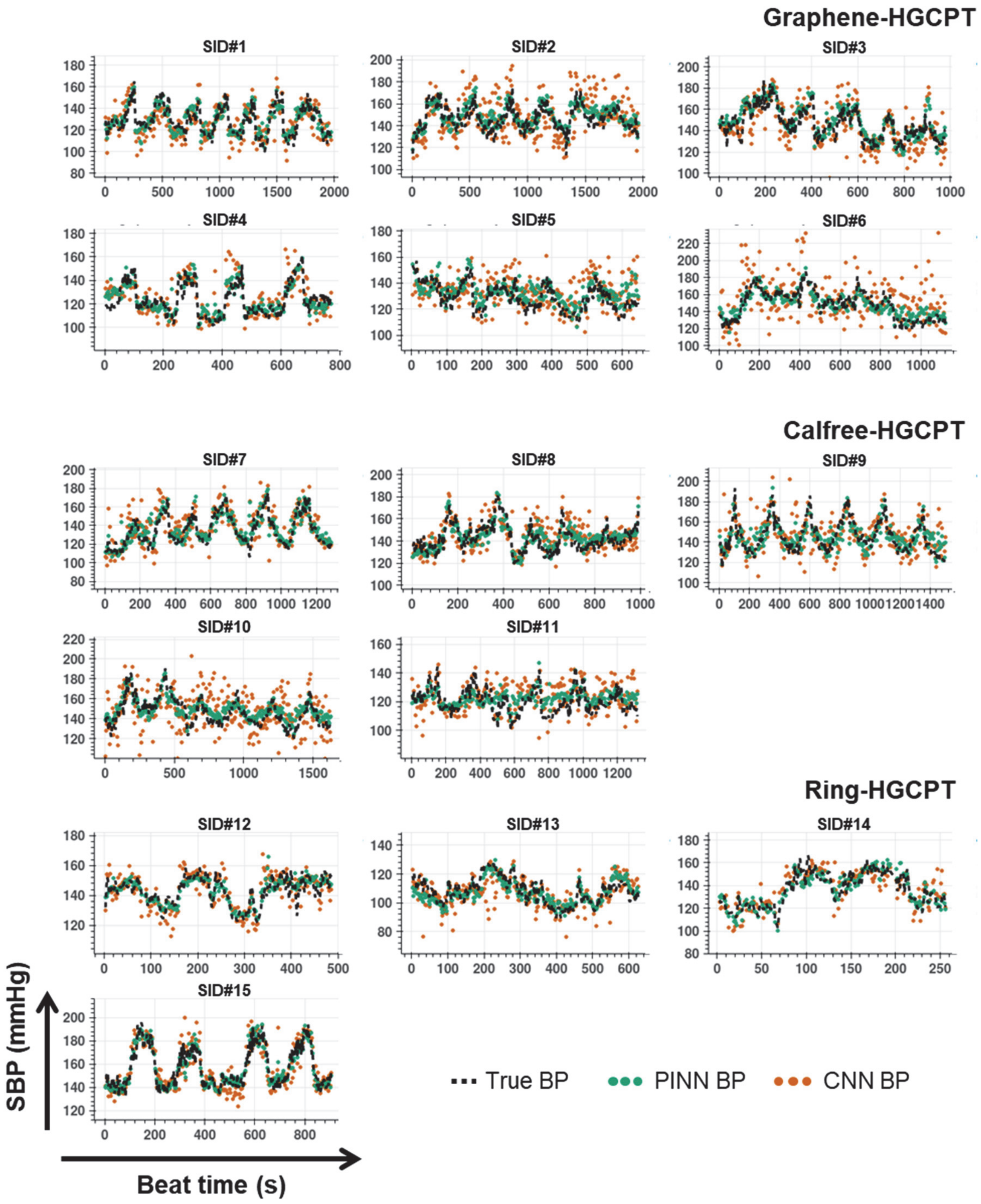

Supplementary Figure 3. Systolic blood pressure (SBP) estimations. The subplots show the estimations for all N=15 participants over three datasets (graphene-HGCPT, calfree-HGCPT, ring-HGCPT). Scattered points show the model estimations for PINN (green) and CNN (orange) in comparison to ground truth BP shown in dashed line (black). The x-axis of the subplots shows the beat times, and the y-axis shows the SBP in mmHg.

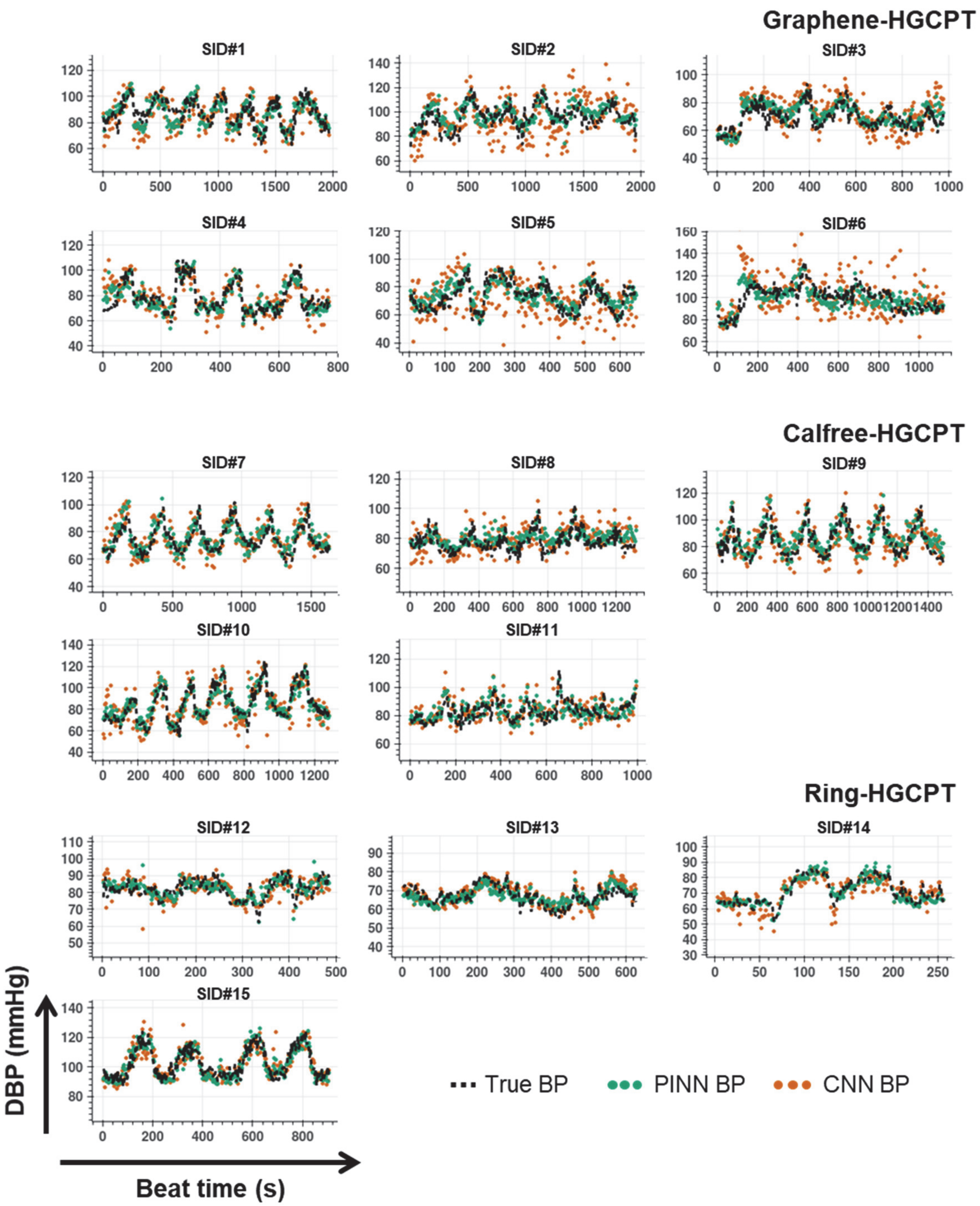

Supplementary Figure 4. Diastolic blood pressure (DBP) estimations. The subplots show the estimations for all N=15 participants over three datasets (graphene-HGCPT, calfree-HGCPT, ring-HGCPT). Scattered points show the model estimations for PINN (green) and CNN (orange) in comparison to ground truth BP shown in dashed line (black). The x-axis of the subplots shows the beat times, and the y-axis shows the DBP in mmHg.

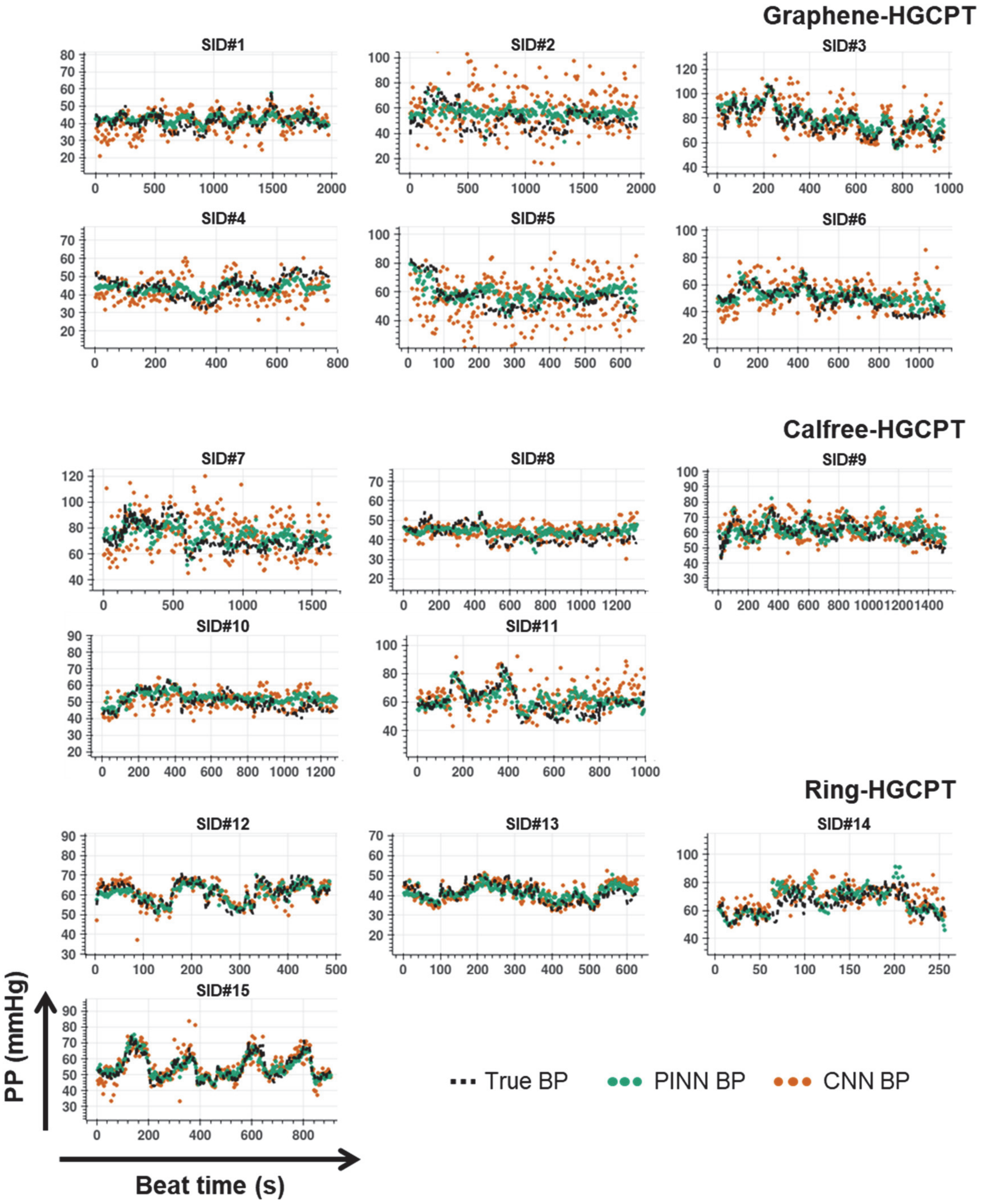

Supplementary Figure 5. Pulse pressure (PP) estimations. The subplots show the estimations for all N=15 participants over three datasets (graphene-HGCPT, calfree-HGCPT, ring-HGCPT). Scattered points show the model estimations for PINN (green) and CNN (orange) in comparison to ground truth BP shown in dashed line (black). The x-axis of the subplots shows the beat times, and the y-axis shows the PP in mmHg.

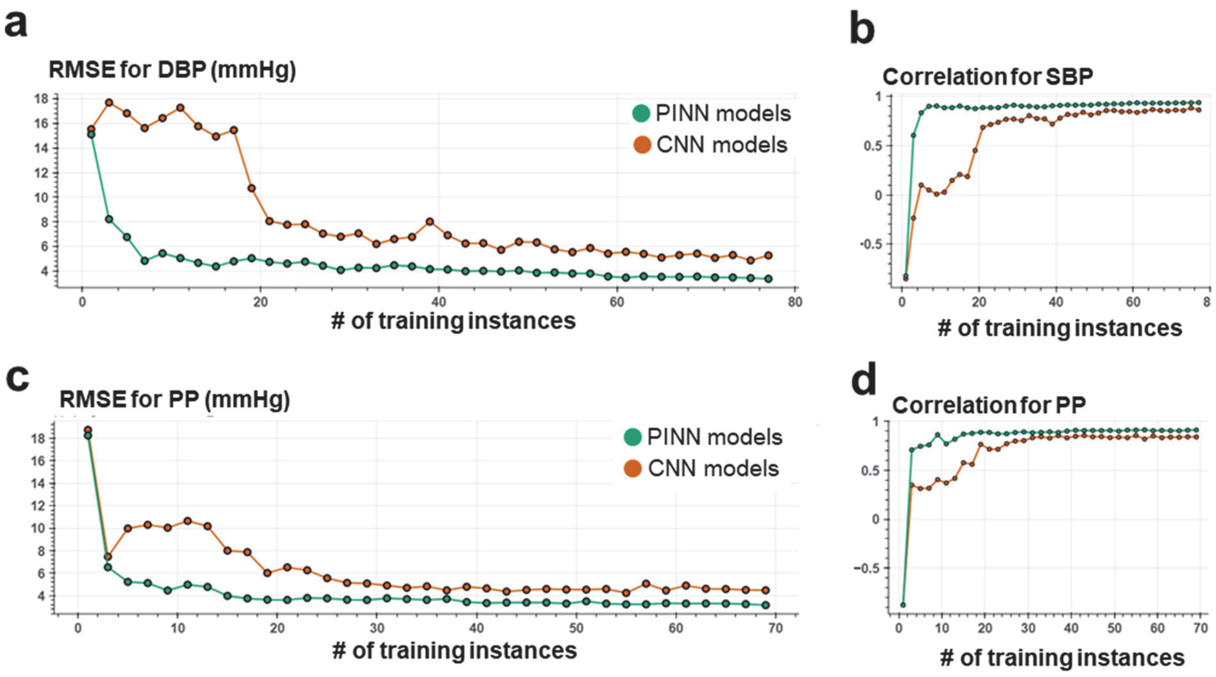

Supplementary Figure 6. PINN and CNN model behavior under increasing number of training instances. (a-b) Root-mean-squared error (RMSE) in mmHg and Pearson's correlation coefficient in estimating DBP for PINN (green) and conventional CNN (orange) models trained with increasing number of training instances. **(c-d)** Root-mean-squared error (RMSE) in mmHg and Pearson's correlation coefficient in estimating PP for PINN (green) and conventional CNN (orange) models trained with increasing number of training instances. PINN models overperforms CNN models independent of the number of labeled instances used for model training. In addition, PINN models show a consistent convergence to a lower error and higher correlation with less than with the gradual increase in the size of the labeled training data.

Supplementary Text

Supplementary Text 1. Blood pressure (BP) is a frequently used cardiovascular (CV) parameter by clinicians to assess the cardiac and circulatory health and associated risk factors and disorders. The conventional BP measurement is based on an oscillometric cuff inflation/deflation, capturing single systolic (SBP), diastolic (DPB) and pulse pressure (PP) values. This results in infrequent readings of BP, and therefore can lead to misdiagnosis caused by inaccurate readings in the presence of measurement biases (e.g., *white coat syndrome*: an artificial rise in BP in medical clinics, while normal at home, *masked hypertension*: BP recorded at normal ranges in clinics, while being high in ambulatory settings)[53]. Moreover, the inflating cuff causes discomfort, preventing the continuous and frequent use to capture useful trends and levels (e.g., normotensive, hypertensive, hypotensive) in BP in ambulatory and nocturnal settings that are necessary to provide accurate CV health assessment and management[16–18]. State-of-the-art wearable cuffless BP technologies address the shortcomings of the cuff, with a trade-off in the fidelity. These technologies leverage non-invasive and unobtrusive modalities (e.g., PPG[54], bioimpedance[3], capacitive[55], ultrasound[56]) to capture certain physiological events and parameters that are relevant to BP. Bioimpedance particularly provides unique advantages over other modalities when used for measuring blood volume changes as the BP pulse wave travels through arteries. These are: (i) due to the use of high-frequency electric current penetration into deep-tissue, bioimpedance sensors capture changes at artery levels rather than skin/capillary level, unlike optical modalities limited with skin-level light penetration[57]; (ii) bioimpedance is insensitive to the variations in participants' skin tone and body-mass index (BMI), unlike optical modalities, where light is attenuated before reaching the arteries for individuals with darker skin tones and higher BMI[57–59]; (iii) bioimpedance provides an area of sensing with proper placement of the skin contact electrodes[60,61], unlike ultrasound[56,62] and radar/Wi-Fi that are highly directional[63].
